# Necrosis of the Lower Jaw from Fumes of Phosphorus

**Published:** 1860-01

**Authors:** 


### SELECTED ARTICLES.

### ARTICLE XI.

*Necrosis of the Lower Jaw from fumes of Phosphorus. Much attention has been recently attracted to this disease, of which we subjoin the reports of cases from different sources.*

FORTUNATELY for us, in this country we do not see so many of the terrible effects arising from the employment of phosphorus in lucifer-match making as are witnessed in France and Germany, where such manufactures are most extensively carried on. Necrosis of the lower jaw is, perhaps the only special result which comes under the notice of the English surgeon, and which does not appear until the teeth first become affected. Diseases of the lungs, in consequence of the manufacture, are with us almost unknown: whilst in Paris and elsewhere many of the workwomen have to leave their employment from what were looked upon as *neglected colds*, terminating in consumption, but which were, in reality, the result of the inhalation of an atmosphere, which Dr. Dupasquier found to consist principally of hypophosphoric acid, probably mixed with small quantities of phosphuretted hydrogen, and possibly the phosphorus itself in the form of vapor. The breath of these people at night becomes luminous. The valuable Report of Dr. Waller Lewis, presented to both Houses of Parliament in 1855, "On the regulation of Noxious Trades and Occupations in France," very clearly shows that bronchitis, often in a very severe form, is a common affection amongst many of the workpeople, and, in proof of this, he furnished abundant evidence from various sources, as well as from personal observation.

Now, although in this country we are free from the worst chases of the disease, it still behoves manufacturers to consider whether, in place of phosphorus, some other chemical substance could not be introduced which would produce the igniting effect desired, without the sickening and unpleasant alliceous odor which invariably arises from this element. Such matches are now being manufactured in Paris by M. Canouil. Amongst other varieties, they are made of chlorate of potass, powdered flint or glass, bichromate of potass, gum or dextrin and water, made into paste. A simple friction produces combustion ; there is no unpleasant smell, because there is no phosphorus, and, what is of considerable importance, their manufacture is not injurious to the workpeople. Much misery and suffering are therefore obviated amongst those who are employed in the factories.

In a former "Mirror," when placing upon record an instance of necrosis of the lower jaw, arising from the fumes of phosphorus, in which Mr. Thomas Wakley removed the bone with success at the Royal Free Hospital, we devoted some attention to the consideration of this subject, and we urged the propriety of the periodical examination of the teeth of those employed in the factories where it was used, so that the spread of its destructive influences might be arrested. We referred to several other cases which had appeared in our "Mirror ;" but Mr. Wakley's was one of the most remarkable we had seen. The patient has since given up his old employment.

Besides necrosis of the jaw and diseases of the lungs, which arise from phosphoric vapors, it has been ascertained by M. Moignot that the women employed in the manufacture of lucifer-matches are very liable to miscarry, and so fully aware have the workmen become of this peculiarity that advantage has been taken of it to procure abortion.

In the case which we now record, the disease did not appear until the gums in contact with the diseased teeth became ulcerated, which produced pain and swelling of the jaw, with ultimate complete death of the entire bone. It is somewhat remarkable, that one condyle, together with the coronoid processes of the rami, should have been withdrawn entire, particularly when we consider the important muscles which are attached to the latter. The phosphoric infiltration remains in the soft structures of the cheeks, thus giving the patient an artificial appearance of health.

Charles T——, aged thirty-nine, a wax-taper dipper, was admitted into the above hospital on the 28th of December, 1858. About thirteen months ago, he complained of much pain and swelling about his lower jaw, commencing in one of the right incisor teeth. Abscesses formed and burst externally through the skin of the cheek. When admitted, his face generally was much swollen, exhibiting the peculiar pasty appearance witnessed in necrosis of the jaw arising from the fumes of phosphorus. Several fistulæ were noticed at the lower margins of the jaw, communicating with dead bone, and giving passage to matter. On opening the mouth, the lower jaw was seen exposed denuded of periosteum, and quite black in color; it was also slightly movable. He had been working for seventeen years at his calling before the ulceration of his gums commenced.

On the 9th of April, chloroform was given by Dr. Martin, and, when anæsthesia was complete, Mr. Coote proceeded to saw through the symphisis of the lower jaw within the mouth; after which, by the aid of a pair of forceps, the left half of the lower jaw was drawn out entire, without its condyle, but with the ascending ramus. The same proceeding was adopted with the right half of the jaw, which came away with equal facility, but with the condyle, which appeared to be healthy. Some hemorrhage necessarily ensued, but it was not great, and spontaneously ceased. For some three or four days after the operation, the patient complained of pain in the face, but it gradually diminished and soon ceased entirely. His health now began to improve, under the use of a liberal diet; his appetite increased, and his strength returned. The investing periosteum of the old bone now began to throw out fresh osseous material, and a new lower jaw was in the process of formation, as has been noticed in other and similar instances.

The pasty appearance of the face, and the puffiness of the cheeks, however, remain and these would seem to be almost the permanent consequences of the affection. He left the hospital a short time back, completely restored in health, and able to speak and articulate with tolerable distinctness.

On examining the bone when cleaned and dried, we found it to be massive, and of nearly double the weight of the healthy bone. It was covered in some places with unhealthy lymph, undergoing osseous transformation.

Ten years ago, there was a patient in this hospital, under Mr. Stanley's care, who was a fellow-workman in the same factory, and whose lower jaw was affected with necrosis arising from the same cause.—*Lancet.*

